# Nano/Micro-Structured ZnO Rods Synthesized by Thermal Chemical Vapor Deposition with Perpendicular Configuration

**DOI:** 10.3390/nano11102518

**Published:** 2021-09-27

**Authors:** Seok Cheol Choi, Do Kyung Lee, Sang Ho Sohn

**Affiliations:** 1 Department of Process Development, LG Electronics, Gumi 39368, Korea; jomi119@hanmail.net; 2 School of Advanced Materials Science and Chemical Engineering, Daegu Catholic University, Gyeongsan 38430, Korea; 3 Department of Physics, Kyungpook National University, Daegu 41566, Korea

**Keywords:** one-dimensional ZnO hierarchical nanostructures, nano/micro-structured ZnO rods, ZnO double-structured rods, thermal chemical vapor deposition, perpendicular configuration, one-step process

## Abstract

Under a one-step process, catalyst-free growth of one-dimensional (1D) ZnO hierarchical nanostructures was performed on ZnO-seeded Si substrate by thermal chemical vapor deposition with a perpendicular setup. The morphological and crystallographic properties of the nano/micro-structured ZnO rods were investigated with varying growth temperature and growth time. X-ray diffraction patterns of 1D ZnO double-structured rods showed the hexagonal wurtzite structure. The morphology and crystal structure of the ZnO double-structured rods were sensitive to the growth temperature and growth time. From Raman scattering and photoluminescence spectra, the orientation and size effects of the ZnO double-structured rods were discussed in relation to growth temperatures and growth times.

## 1. Introduction

ZnO nanostructures have attracted attention due to their unique electrical and optical properties for potential applications to nanolasers [1], light-emitting devices [2], photosensors [3], gas sensors [4], catalysis [5], and solar cells [6]. In particular, for catalysis and photocatalysis applications, specific surface areas of ZnO nanostructures can be enhanced by controlling their shapes and morphologies or by controlling the distribution of ZnO nanostructures. Hence, it is prerequisite to synthesize ZnO nanostructures with unique spatial architectures and specific surface area. Low-dimensional ZnO hierarchical architectures can offer the possibility to overcome the problem and the chance for development of novel functional devices. Various wet chemical methods, such as the hydrothermal method [7,8,9,10], modified sol–gel method [11], microwave method [12], solvothermal method [13], and chemical bath deposition [14], have been developed to synthesize the ZnO hierarchical nanostructure.

For chemical vapor deposition (CVD), as one of the dry methods, some literature on the fabrication and characterization of ZnO hierarchical architectures was reported. Most CVD methods for ZnO hierarchical nanostructures are based on multi-step fabrication process. For example, Wang et al. [15] reported that Al-doped ZnO hierarchical nanostructures, which are the structures of nanorod/thin cap/nanorod, were successfully synthesized by CVD with a ZnO seed layer. Although the growth temperature was found to be the major factor for the changes in morphology of the Al-doped ZnO hierarchical nanostructures, the heating temperature varied with a two-step during the growth. Devika et al. [16] reported the synthesis of a vertically aligned ZnO hierarchical nanostructure by CVD using a three-step process. However, a multi-step fabrication process has the disadvantage of manufacturing cost.

In previous work, two-dimensional ZnO nanostructures showing various nanoarchitectures were successfully synthesized by thermal CVD with parallel and perpendicular setups [17]. In a perpendicular configuration, depending on growth temperature, ZnO nanostructures grown on Au-coated Si substrates exhibited nanorods and nanosaws, and the growth of ZnO nanostructures on bare Si substrates gave rise to nanosheets, nanoplates, and nanobelts, resulting in various shapes and morphologies. Thus, thermal CVD growth of ZnO nanostructures in a perpendicular setup under specific growth conditions has the potential for fabrication of unique nanoarchitectures. 

The main objective of our work is to attempt to fabricate a low-dimensional ZnO hierarchical nanostructure with the help of the perpendicular-configurated CVD system with a one-step process. It has been well known that the surface energy of the ZnO seed layer influences greatly the growth of ZnO nanostructures [18]. The surface of a hydrophobic ZnO seed layer may induce spatially nonuniform aggregation of the ZnO nuclei at the initial growth of ZnO nanostructures. In addition, it was reported that ZnO microcrystals agglomerate very quickly to form a smooth and closed layer at the initial stages under a high-temperature CVD-based growth process [19].

In this work, one-dimensional (1D) ZnO double-structured rods, which are the structure with nanorods on top of microrods, were prepared by thermal CVD with a perpendicular setup on the hydrophobic ZnO-seeded Si substrate, varying growth temperature from 700 to 900 °C and growth time from 20 to 60 min. The morphological and structural properties and size effects of the ZnO double-structured rods were investigated with respect to growth temperatures and growth times. 

## 2. Experiments

A thermal CVD system with perpendicular setup was used for the synthesis of 1D ZnO double-structured rods. A schematic of the thermal CVD with perpendicular setup is provided in the Supplementary Materials (Figure S1). Prior to growth of ZnO double-structured rods, about 100 nm-thick ZnO thin films were initially deposited on Si substrate as a seed layer by a conventional radio frequency sputtering method using a ZnO target (99.99%, Nichia, Japan) at substrate temperatures of 250 °C. The surface contact angle of the ZnO seed layer is represented in the Supplementary Materials (Figure S2). For the growth of 1D ZnO double-structured rods, a powder mixture of ZnO (99.9%, Sigma-Aldrich, Saint Louis, MO, USA) and graphite powder (99.9%, Sigma-Aldrich, Saint Louis, MO, USA) at a 1:1 ratio in weight was used as an evaporation source. The particle size of ZnO and graphite powders was below 5µm and 45µm, respectively. The growth temperature and growth time of the ZnO double-structured rods were changed from 700 to 900 °C and from 20 to 60 min, respectively. During the growth of ZnO double-structured rods, a mixed gas of Ar and O_2_ was introduced in the reactor, with constant Ar/O_2_ flow of 120/0.1 sccm. 

The shapes and morphologies of the ZnO double-structured rods were observed using a field emission scanning electron microscope (FE-SEM, S-4800, Hitachi, Tokyo, Japan). Quantitative analysis for chemical composition of ZnO double-structured rods was performed by using an electron probe micro-analyzer (EPMA, EPMA-1600, Shimadzu, Kyoto, Japan) and energy dispersive X-ray spectroscopy (EDS, S-4800, Hitachi, Tokyo, Japan). The crystallinity, crystal structure, and crystal orientation of ZnO double-structured rods were investigated by X-ray diffraction (XRD, X’Pert MPD, PANalytical, Almelo, Netherlands) patterns measured with Cu-Kα radiation. Raman scattering measurements were performed at room temperature using a Raman spectrometer (SPEX 1403, Horiba, Kyoto, Japan) with a 532 nm line of an Ar+ laser source. The laser beam spot size was about 2 μm. The photoluminescence (PL, LabRam HR, Horiba, Kyoto, Japan) characteristics of ZnO double-structured rods were measured at room temperature using a 325 nm wavelength He–Cd Laser as the excitation source. 

## 3. Results and Discussion

**Figure 1:**
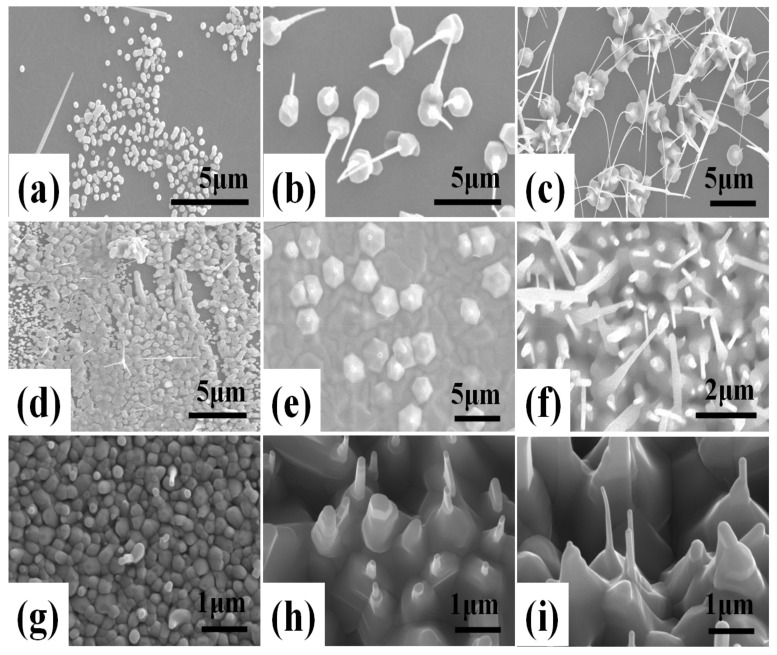
FE-SEM images of ZnO double-structured rods grown on ZnO-coated Si substrates by thermal CVD with perpendicular setup as functions of growth temperature and growth time: at 700 °C for (**a**) 20 min, (**b**) 40 min, and (**c**) 60 min; at 800 °C for (**d**) 20 min, (**e**) 40 min, and (**f**) 60 min; at 900 °C for (**g**) 20 min, (**h**) 40 min, and (**i**) 60 min.

Figure 1 shows FE-SEM images of ZnO double-structured rods grown on hydrophobic ZnO-coated Si substrates under different growth temperatures and growth times by thermal CVD with a perpendicular setup. For a growth time of 20 min, ZnO nanocrystallites showed an increase in width from about 100 to 250 nm with increasing growth temperature. Moreover, the increase in growth temperature led to the change in the growth mode of ZnO nanocrystallite from an island to a layered structure, as shown in Figure 1a,d,g. The increase in the nanocrystallite size and the change in growth mode may arise from the increase in activation energy for the nucleation and growth of ZnO nanocrystallites due to the increasing growth temperature. As shown in Figure 1b,e,h, at a growth time of 40 min, ZnO double-structured rods could be observed, which consist of microrods and nanorods. In Figure 1b, ZnO microrods with a width ranging from 1.5 to 2.5 µm were vertically grown on the substrate. Moreover, ZnO nanorods grown on the microrods were randomly oriented with the slightly straight form. As shown in Figure 1e, compared to the growth temperature of 700 °C, ZnO microrods grown at 800 °C began to aggregate and increased in width. In addition, short ZnO nanorods similar to nanodots were grown on the microrods. At a growth temperature of 900 °C, ZnO microrods were aggregated to each other and the widths and lengths of ZnO nanorods further increased. For a growth time of 60 min, ZnO nanorods grown at a growth temperature of 700 °C significantly increased in length, resulting in bending and interconnection. ZnO nanorods grown at 800 °C showed a remarkable increase in widths and lengths, representing random orientation. At a growth temperature of 900 °C, ZnO nanorods grew slightly, having widths of about 100 nm and lengths of about 1.4 µm. In the case of ZnO nanostructures grown by thermal CVD, Zn vapor can be formed by the carbothermal reduction process from the powder mixture of ZnO and C. Then, the Zn and O vapors form liquid droplets, leading to the saturation of droplets and the subsequent formation of ZnO nanocrystallites. ZnO nanowires and microrods are grown onto the ZnO nanocrystallites. Therefore, the formation of ZnO nanorods and microrods can be attributed to the vapor–liquid–solid (VLS) mechanism via a self-catalytic process [20]. Accordingly, the width and length of ZnO double-structured rods are dependent on the growth temperature and growth time, due to the VLS mechanism via a self-catalytic process, resulting in the morphology change of ZnO double-structured rods.

**Figure 2:**
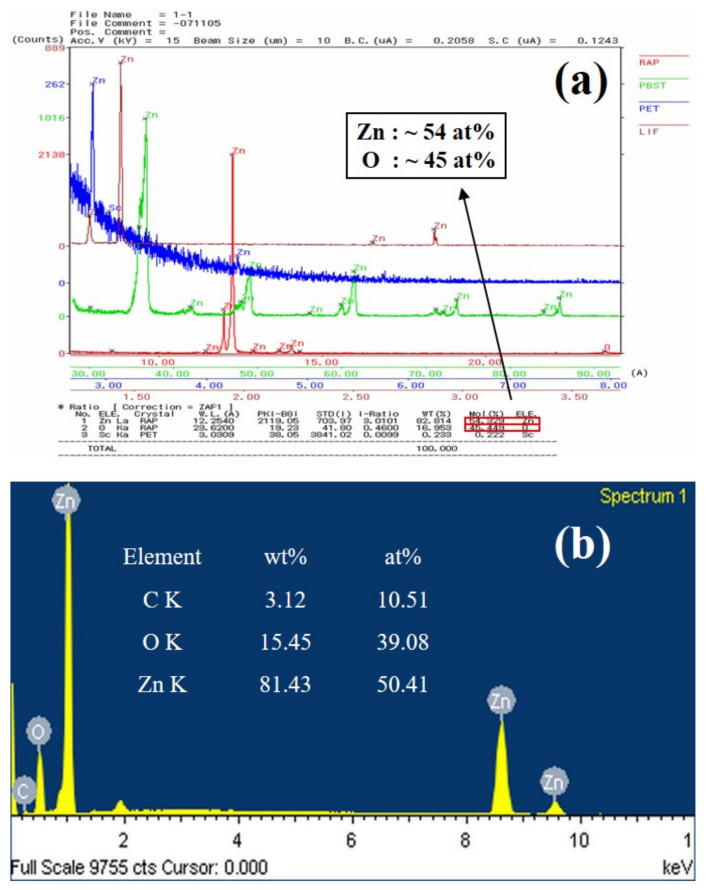
(**a**) EPMA and (**b**) EDS spectra of ZnO double-structured rods grown at a growth temperature of 700 °C for a growth time of 60 min.

Figure 2 shows EPMA and EDS spectra of ZnO double-structured rods grown at a growth temperature of 700 °C for a growth time of 60 min. From the EPMA result, we observed that ZnO double-structured rods were only composed of Zn and O (with the exception of a few Sc elements due to an impurity under measurements), as shown in Figure 2a. The stoichiometry of ZnO double-structured rods can be represented by the atomic percentages of Zn and O, which are about 54% and 45%, respectively. Moreover, as shown in Figure 2b, the stoichiometry of ZnO double-structured rods from the EDS spectrum was similar to that of the EPMA result, indicating oxygen-deficient ZnO double-structured rods.

**Figure 3:**
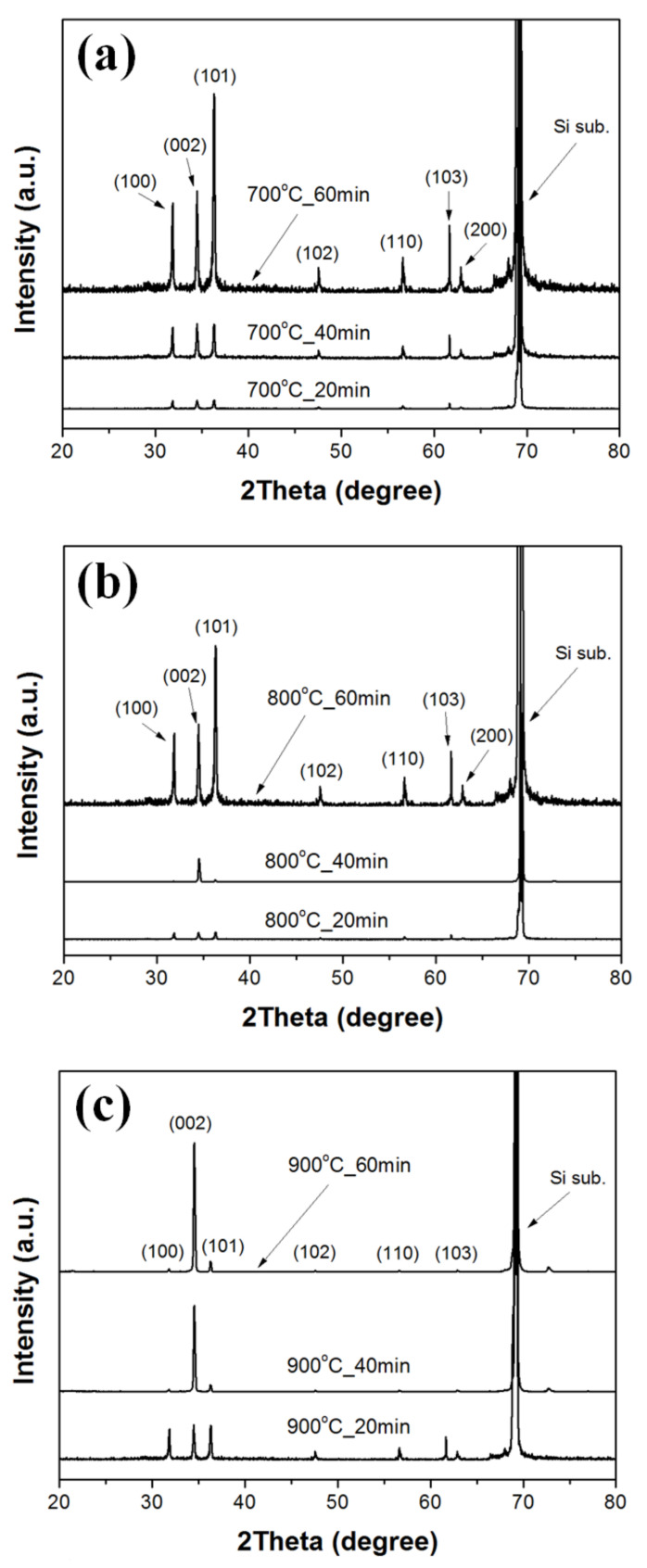
XRD patterns of the ZnO double-structured rods grown on ZnO-coated Si substrates at a growth temperature of (**a**) 700, (**b**) 800, and (**c**) 900 °C.

Figure 3 shows XRD patterns of ZnO double-structured rods grown on ZnO-coated Si substrates under different growth temperatures and growth times. For all the samples, we observed a peak at about 69.16° due to the Si substrate. All diffraction peaks in the XRD patterns of ZnO double-structured rods corresponded to the hexagonal wurtzite structure of ZnO (Joint Committee on Powder Diffraction Standards (JCPDS) no. 89-1397). Except for the ZnO double-structured rods grown at a growth temperature of 800 °C for a growth time of 40 min, the diffraction patterns of the ZnO double-structured rods grown at 700 °C and 800 °C were similar to each other and indicated the powder-like patterns of ZnO, implying a random orientation of ZnO double-structured rods. The diffraction peak of the (002) plane for the ZnO double-structured rods grown at 800 °C for 40 min was clearly observed due to vertically oriented ZnO double-structured rods. For the ZnO double-structured rods grown at a growth temperature of 900 °C, the diffraction peak intensity of the (002) plane was significantly higher than those of the (100) and (101) planes, thereby implying a preferred orientation in the c-axis. Moreover, the diffraction peak intensity of the (002) plane increases with increasing growth time, enhancing the preferred orientation in the c-axis. These XRD results were in good agreement with the FE-SEM observations, as shown in Figure 1.

**Figure 4:**
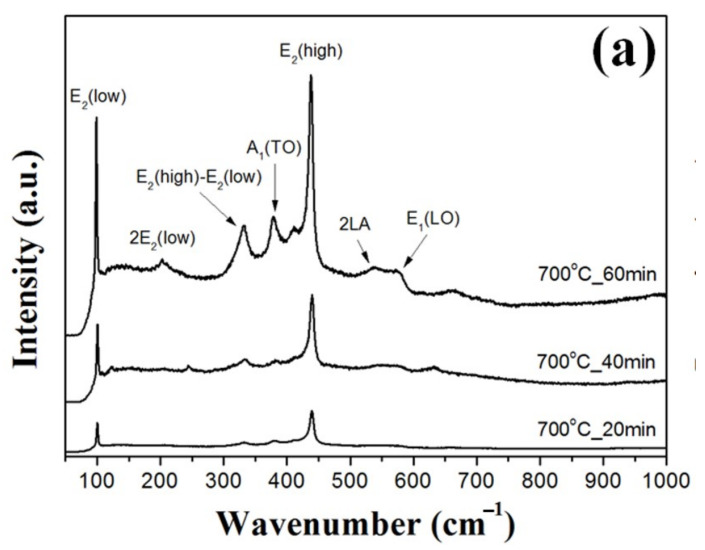

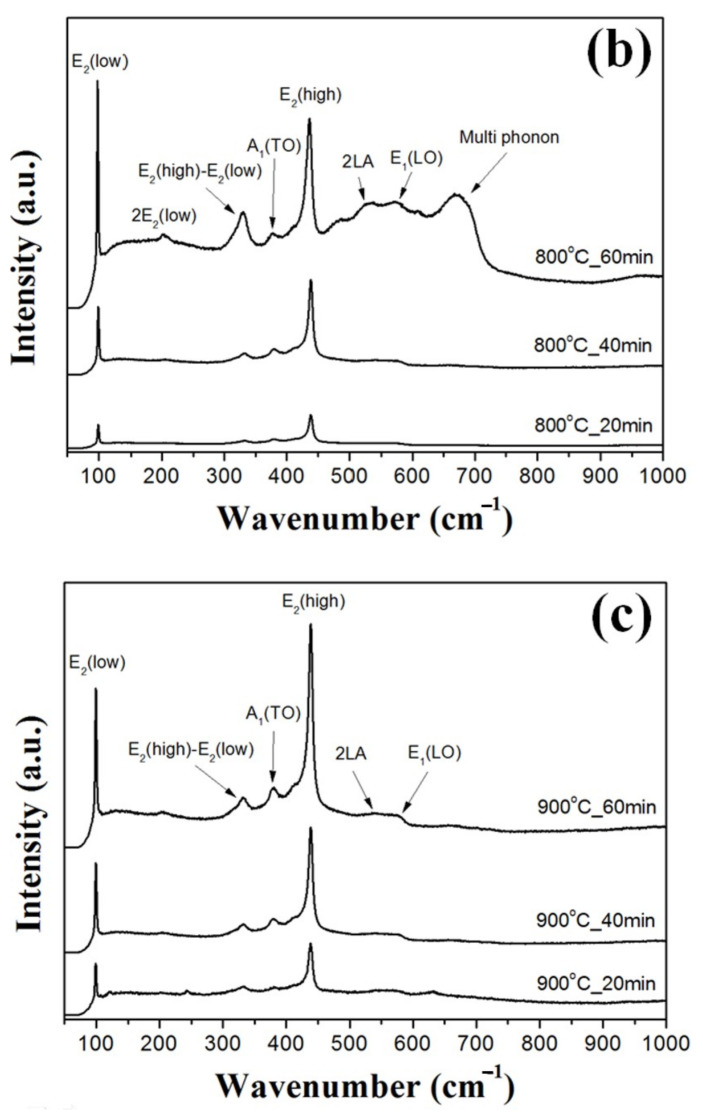
Raman scattering spectra of the ZnO double-structured rods grown at a growth temperature of (**a**) 700, (**b**) 800, and (**c**) 900 °C.

Figure 4 shows Raman scattering spectra of the ZnO double-structured rods grown as functions of growth temperature and growth time. From the XRD results, the ZnO double-structured rods showed the hexagonal wurtzite structure, which belongs to the space group C46v (P63mc). The optical phonons at the Γ point of the Brillouin zone belong to the following irreducible representations [21]:

Γ_opt_ = A_1_ + 2B_1_ + E_1_ + 2E_2_, (1)

where both A_1_ and E_1_ are polar modes, and split into transverse optical (TO) and longitudinal optical (LO) components with different frequencies. The E_2_ mode has two modes, including low- and high-frequency phonons (E_2_ (low) and E_2_ (high)), which are consistent with the vibrations of the heavy Zn sublattice and oxygen atoms, respectively [22]. In Figure 4, Raman scattering peaks positioned at 101, 330, 382, 436, 545, 586, and 660 cm^−1^ correspond to the E_2_ (low), E_2_ (high)–E_2_ (low), A_1_ (TO), E_2_ (high), 2LA, E_1_ (LO), and multi-phonon modes, respectively. For the ZnO double-structured rods grown at 700 °C for 40 min, the E_2_ (high) mode was strongly observed compared to other modes due to some vertically aligned ZnO double-structured rods. However, at the same growth temperature, the ZnO double-structured rods grown for 60 min showed the increase in the peak intensities at all the vibrational modes due to many randomly aligned structures, including bended ZnO nanowires. In addition, the peak intensity at the A_1_ (TO) mode was higher than that at the E_2_ (high)–E_2_ (low) mode, which may be originated from the decrease in widths and random orientation of bended nanowires. As shown in Figure 4b, the ZnO double-structured rods grown at 800 °C for 40 min indicated that the peak intensity at the E_2_ (high) mode increased due to the aggregated ZnO microrods. For the ZnO double-structured rods grown at 800 °C for 60 min, the peak intensities at all the vibrational modes increased due to many randomly aligned nanostructures. Moreover, unlike the ZnO double-structured rods grown at 700 °C for 60 min, the ZnO double-structured rods grown at 800 °C for 60 min represented that the peak intensity at the A_1_ (TO) mode was lower than that at the E_2_ (high)–E_2_ (low) mode because of the randomization effects of the nanorods. Raman peak intensities at all the vibrational modes for the ZnO double-structured rods grown at 900 °C for 40 min increased due to the tapered ZnO microrods and the ZnO nanorods with small widths. The ZnO double-structured rods grown at 900 °C for 60 min showed that the peak intensities at all the vibrational modes also increased due to some ZnO nanowires and many tapered ZnO microrods. However, similar to the ZnO double-structured rods grown at 700 °C for 60 min, the ZnO double-structured rods grown at 900 °C for 60 min showed that the peak intensity at the A_1_ (TO) mode was higher than the peak intensity at the E_2_ (high)–E_2_ (low) mode because of the randomization and the width reduction of the nanorods.

**Figure 5:**
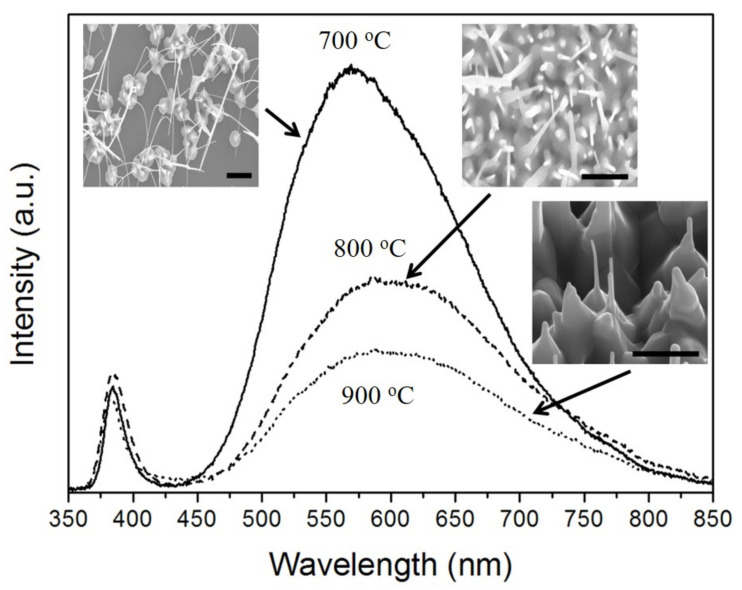
PL spectra of the ZnO double-structured rods grown for a growth time of 60 min under different growth temperatures. The insets correspond to Figure 1c,f,i.

Figure 5 shows the PL spectra of the ZnO double-structured rods grown for 60 min under different growth temperatures. A sharp UV emission centered at about 380 nm and a broad visible emission centered at about 570 nm were observed. The UV and visible emission are generally due to a near-band edge and deep-level recombination of ZnO, respectively [23,24]. Among the visible emissions, the green emission is commonly ascribed to oxygen vacancies [25,26]. The PL spectra showed that the visible emission intensity decreased with an increase in growth temperature, implying a decreasing intensity ratio between UV and visible emission. Although the composition of the ZnO double-structured rods is oxygen deficient, as mentioned in Figure 2, there are a few composition variations of the ZnO nano/microrods with varying growth temperature. Thus, a decrease in the intensity ratio between UV and visible emission can be considered by the surface effects of the ZnO nanostructures [27]. Because the increase in surface area of the ZnO double-structured rods gives rise to enhancing the recombination of trapped holes with free electrons in the ZnO double-structured rods, the width of the ZnO double-structured rods increases with increasing growth temperature, as shown in the insets of Figure 5, resulting in the decreasing visible emission intensity.

## 4. Conclusions

By using thermal CVD with a perpendicular setup, we synthesized the 1D ZnO double-structured rods on hydrophobic ZnO-coated Si substrates through the VLS mechanism via a self-catalytic process. In FE-SEM analysis, the morphology change of the ZnO double-structured rods could be obtained by controlling growth temperature and growth time. With an increase in growth temperature at a growth time of 60 min, the width of the ZnO double-structured rods increased. From XRD results, the ZnO double-structured rods had hexagonal wurtzite structure. With a growth time of 60 min, the ZnO double-structured rods grown at growth temperatures of 700 and 800 °C exhibited the random orientation, but the ZnO double-structured rods grown at 900 °C indicated the preferred orientation in the c-axis. The Raman spectra show that A_1_ (TO) mode increased with increasing growth time under the same growth temperature, originating in the randomization and the width reduction of the nanorods in the ZnO double-structured rods. The surface area of the ZnO double-structured rods increased with increasing growth temperature, resulting from the increase in vibrational modes of Raman scattering and the decrease in visible emission of PL spectra. From the device application point of view, because the stability and reliability of the ZnO double-structured rods are crucial factors in device performance, further investigations on their mechanical and chemical stability are needed.

## Data Availability

Not applicable.

## Supplementary Materials

The following are available online at https://www.mdpi.com/article/10.3390/nano11102518/s1, Figure S1: Schematic of thermal CVD with perpendicular setup. Figure S2: Water contact angle on the surface of ZnO-seeded Si substrate.
