# Supplementary material for: Nano/Micro-Structured ZnO Rods Synthesized by Thermal Chemical Vapor Deposition with Perpendicular Configuration
Source: Nanomaterials (Basel). 2021 Sep 27;11(10):2518. doi: 10.3390/nano11102518 (PMC8538317; doi:10.3390/nano11102518)
Supplement: Supplementary file 1 [file nanomaterials-11-02518-s001.zip › nanomaterials-1372301-supplementary.pdf]

Supplementary Material

Nano/Micro-Structured ZnO Rods Synthesized by Thermal Chemical Vapor Deposition with Perpendicular Configuration

Seok Cheol Choi [1], Do Kyung Lee [2,*] and Sang Ho Sohn [3,*]

1 Department of Process Development, LG Electronics, Gumi 39368, Korea; jomi119@hanmail.net
2 School of Advanced Materials Science and Chemical Engineering, Daegu Catholic University, Gyeongsan 38430, Korea
3 Department of Physics, Kyungpook National University, Daegu 41566, Korea
* Correspondence: dokyung@cu.ac.kr (D.K.L.); shsohn@knu.ac.kr (S.H.S.)

For the synthesis of nano/micro-structured ZnO rods, thermal chemical-vapor deposition (CVD) system with perpendicular setup was used, seen in Figure S1. The experimental setup was vertically positioned by reversely loading the ZnO-seeded Si substrate at the top of an alumina holder. The distance between the substrate and ZnO+C precursor was fixed at about 4 mm.

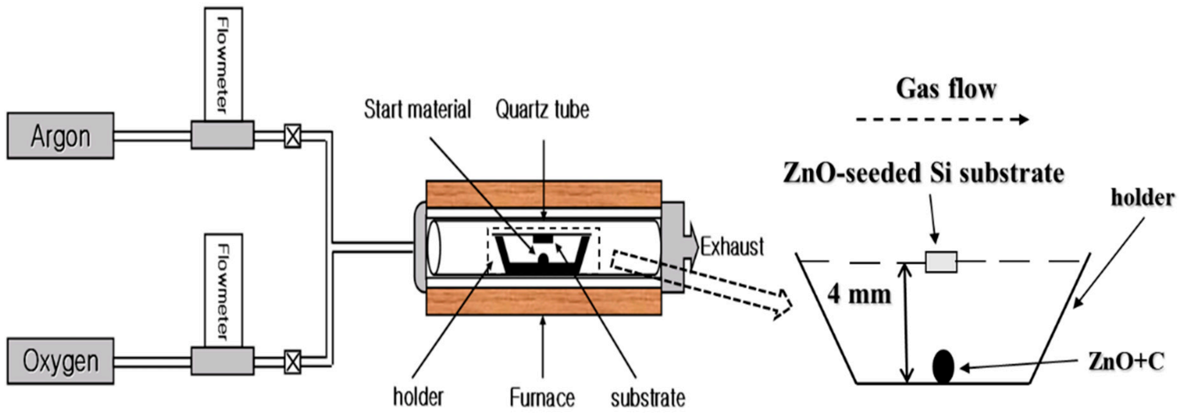

Figure S1. Schematic of thermal CVD with perpendicular setup.

Figure S2 shows the water contact angle on the surface of ZnO-seeded Si substrate. The contact angle of ZnO-seeded Si substrate surface was measured using the contact angle measurement equipment (KRUSS, DSA100, Hamburg, Germany) at room temperature. Surface energy of ZnO-seeded Si substrate was calculated by using the Neumann method [S1]. As shown in Figure S2, the contact angle of ZnO-seeded Si substrate surface was 92 ° and the corresponding surface energy was 20.9 dyne/cm, indicating a slight hydrophobic surface. These are similar to that reported by Davea et.al. [S2].

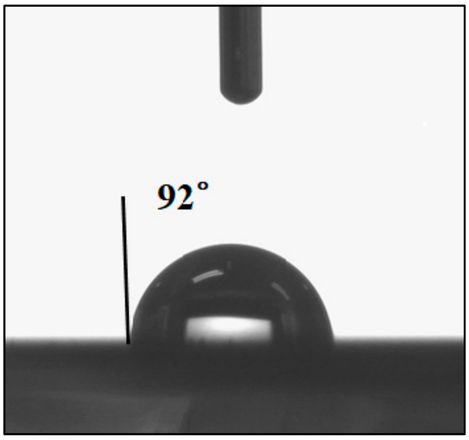

Figure S2. Water contact angle on the surface of ZnO-seeded Si substrate.

References

[S1] Kwok, D.Y.; Lam, C.N.C.; Li, A.; Zhu, K.; Wu, R.; Neumann, A.W.; Low-rate dynamic contact angles on polystyrene and the determination of solid surface tensions, *Polym. Eng. Sci*. **1998**, 38, 1675–1684.

[S2] Davea, P. Y.; Patela, K. H.; Chauhana, K. V.; Chawlab, A. K.; Rawala, S. K.; Examination of zinc oxide films prepared by magnetron sputtering, *Procedia Technology* **2016,** 23, 328–335.
